# Mastl kinase, a promising therapeutic target, promotes cancer recurrence

**DOI:** 10.18632/oncotarget.2565

**Published:** 2014-12-22

**Authors:** Ling Wang, Vivian Q. Luong, Peter J. Giannini, Aimin Peng

**Affiliations:** ^1^ Department of Oral Biology, College of Dentistry, University of Nebraska Medical Center, Lincoln, NE 68583

**Keywords:** cancer, chemotherapy, recurrence, Mastl, Greatwall

## Abstract

Mastl kinase promotes mitotic progression and cell cycle reentry after DNA damage. We report here that Mastl is frequently upregulated in various types of cancer. This upregulation was correlated with cancer progression in breast and oral cancer, poor patient survival in breast cancer, and tumor recurrence in head and neck squamous cell carcinoma. We further investigated the role of Mastl in tumor resistance using cell lines derived from the initial and recurrent tumors of the same head and neck squamous cell carcinoma patients. Ectopic expression of Mastl in the initial tumor cells strongly promoted cell proliferation in the presence of cisplatin by attenuating DNA damage signaling and cell death. Mastl knockdown in recurrent tumor cells re-sensitized their response to cancer therapy *in vitro* and *in vivo*. Finally, Mastl targeting specifically potentiated cancer cells to cell death in chemotherapy while sparing normal cells. Thus, this study revealed that Mastl upregulation is involved in cancer progression and tumor recurrence after initial cancer therapy, and validated Mastl as a promising target to increase the therapeutic window.

## INTRODUCTION

Understanding the molecular mechanism of cancer progression is imperial to cancer prevention and early diagnosis. Elucidating the cellular pathways that render tumor cells resistant to cancer treatment will propel the development of more effective cancer therapeutics. As uncontrolled cell proliferation represents a unifying feature of cancer, it is not surprising that cell division in mitosis has been shown to play a critical role in cancer progression, whereas anti-mitotic drugs have been proven valuable in cancer therapy [1, 2]. For example, Plk1, Aurora A, and Aurora B are serine/threonine kinases that regulate multiple aspects of mitotic progression. These kinases have been shown to be upregulated in various types of cancer, consistent with a large body of evidence that indicated the oncogenic activity of these kinases in established human cell lines and animal models. To date, a number of small molecule inhibitors of Plk1 and Aurora kinases are under clinical development for cancer therapy [3].

Compared to Plk1 and Aurora kinases, microtubule-associated serine/threonine kinase like (Mastl), another protein kinase required for mitotic regulation, is much less studied. First identified in *Drosophila* and then functionally characterized in *Xenopus* egg extracts as the Greatwall (Gwl) kinase, Mastl is known to be activated through its mitotic phosphorylation catalyzed by Cdk1, Mastl itself, and possibly other kinases [4–8]. It has been subsequently discovered that Mastl regulates mitotic entry and maintenance by inhibiting PP2A/B55δ, the principal protein phosphatase complex that dephosphorylates CDK substrates [9–16]. The mechanism of PP2A/B55δ inhibition by Mastl has been attributed to endosulfine and its related family member, cAMP-regulated phosphoprotein 19kDa, which specifically bind and inhibit PP2A/B55δ when they are phosphorylated by Mastl [14, 16]. While delineated largely in *Xenopus* egg/oocyte systems, the function of Mastl is well-conserved in human cells. Disruption of Mastl expression in human cells led to defects in chromosome condensation, separation, and many other aspects of mitotic progression [9, 17, 18].

Interestingly, our recent study showed that Mastl also functions as a regulator of the DNA damage response (DDR), a cellular surveillance mechanism [19]. DNA damage is frequently induced in cells by endogenous, metabolic products, as well as environmental agents. DNA damage quickly activates the DDR that encompasses DNA repair, cell cycle checkpoint, and cell death [20–22]. It has been well established that the DDR is critically involved in cancer progression and therapy. Mutations in many DDR genes can lead to cancer predisposition, indicating an important role of the DDR in tumor suppression [23, 24]. Recent studies in various types of somatic cancers have also shown that the DDR is generally activated in pre-cancerous cells as an anti-cancer barrier; overcoming the DDR barrier is a crucial step in the progression of cancer [25–28]. Moreover, studies of the DDR process may reveal valuable insights into cancer treatment, especially in radiation and chemotherapy using genotoxic agents. These treatments efficiently kill cancer cells in some cases, but the outcome is often restricted in others due to cancer resistance [29, 30]. In previous studies, we reported that depletion of Mastl from interphase *Xenopus* egg extracts augmented DNA damage signaling and impeded checkpoint recovery [19]. We further showed that the involvement of Mastl in the DDR is distinct from, but related to that of Plk1 [31].

Based on the function of Mastl, as demonstrated in *Xenopus* and other experimental systems, we speculated that Mastl may be involved in the pathophysiology of human diseases, particularly cancer. In this study, we discovered that Mastl upregulation is a relatively common event in various forms of human cancer. The relevance of Mastl upregulation to cancer progression and resistance was established. Using a panel of cell lines that were clinically derived from the initial and recurrent tumors of the same patients, our study linked Mastl to tumor recurrence, and validated Mastl as an effective and potentially specific target for cancer therapy.

## RESULTS

### Overexpression of Mastl in cancer

**Figure 1:**
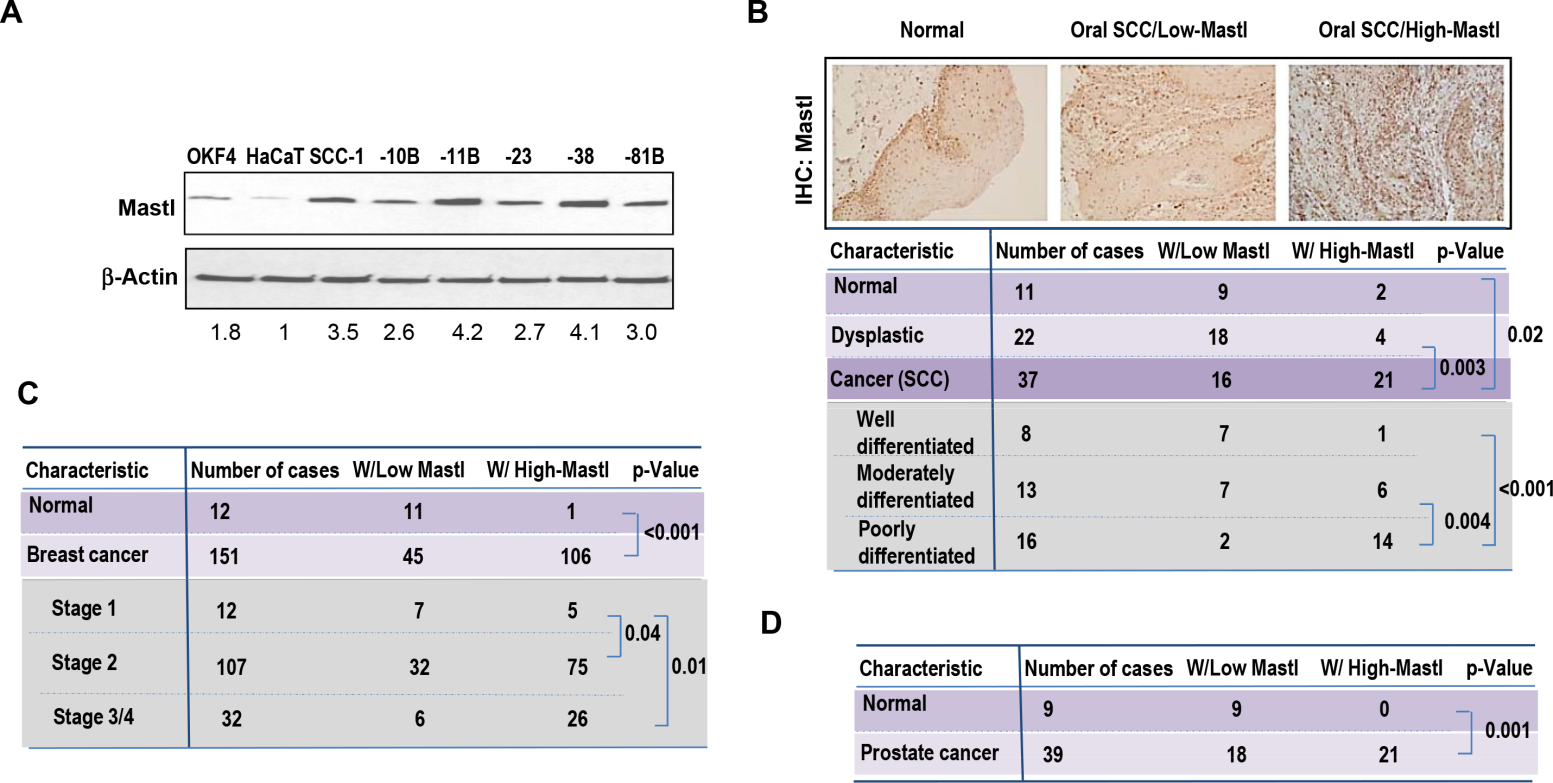
Overexpression of Mastl in cancer **(A)** Mastl expression in a panel of UM-SCC cell lines, and two non-tumorigenic keratinocyte cell lines, HaCaT and OKF4. Immunoblotting of Mastl and β-Actin is shown. The band intensity was measured and the Mastl/β-Actin ratio shown below. **(B)** Normal, dysplastic and SCC oral tissues were analyzed by immunohistochemistry (IHC) for Mastl expression as described in Materials and Methods. Representative images of Mastl IHC are shown on the top panel. Statistical significance was determined by Student's t-test. **(C)** Expression of Mastl in breast cancer tissues and adjacent normal tissues was examined by IHC as in panel B. **(D)** Expression of Mastl in prostate cancer tissues and adjacent normal tissues was examined by IHC as in panel B.

Despite recent studies in *Drosophila*, *Xenopus*, and mammalian cells that revealed the cellular function of Mastl, it remained unclear whether and how Mastl is involved in cancer. Such a notion is interesting given the role of Mastl in promoting mitotic entry and DNA damage checkpoint recovery. We analyzed the protein level of Mastl in a panel of head and neck squamous cell carcinoma (SCC) cell lines to determine if the level of Mastl is altered in cancer. Compared to two normal control cell lines, several cancer cell lines exhibited higher levels of Mastl (Fig. 1A). Harboring the highest Mastl expression among these cell lines are UM-SCC-11B and UM-SCC-38, both of which were shown in our previous study to be highly resistant to cisplatin treatment [32].

We then analyzed the expression of Mastl in archival oral squamous cell carcinoma tissue samples that were collected and histopathologically diagnosed in the UNMC College of Dentistry Oral and Maxillofacial Pathology Laboratory. Normal and dysplastic oral tissues collected in the same laboratory were used as controls. Interestingly, more than half of all oral cancer tissues displayed elevated levels of Mastl expression, which represented a statistically significant difference when compared to the normal or dysplastic tissues (Fig. 1B). Further analysis revealed a strong correlation between Mastl upregulation and more aggressive characteristics of cancer (Fig. 1B). In addition to oral cancer, a similar fashion of clinical involvement of Mastl may account for other types of cancer. For example, in both breast and prostate cancer cases, Mastl upregulation was frequently observed in cancerous, but not normal tissues (Fig. 1C & 1D). Furthermore, the elevated expression of Mastl correlated with a more advanced clinical stage of breast cancer (Fig. 1C).

### Mastl upregulation correlates with poor patient survival and tumor recurrence

**Figure 2:**
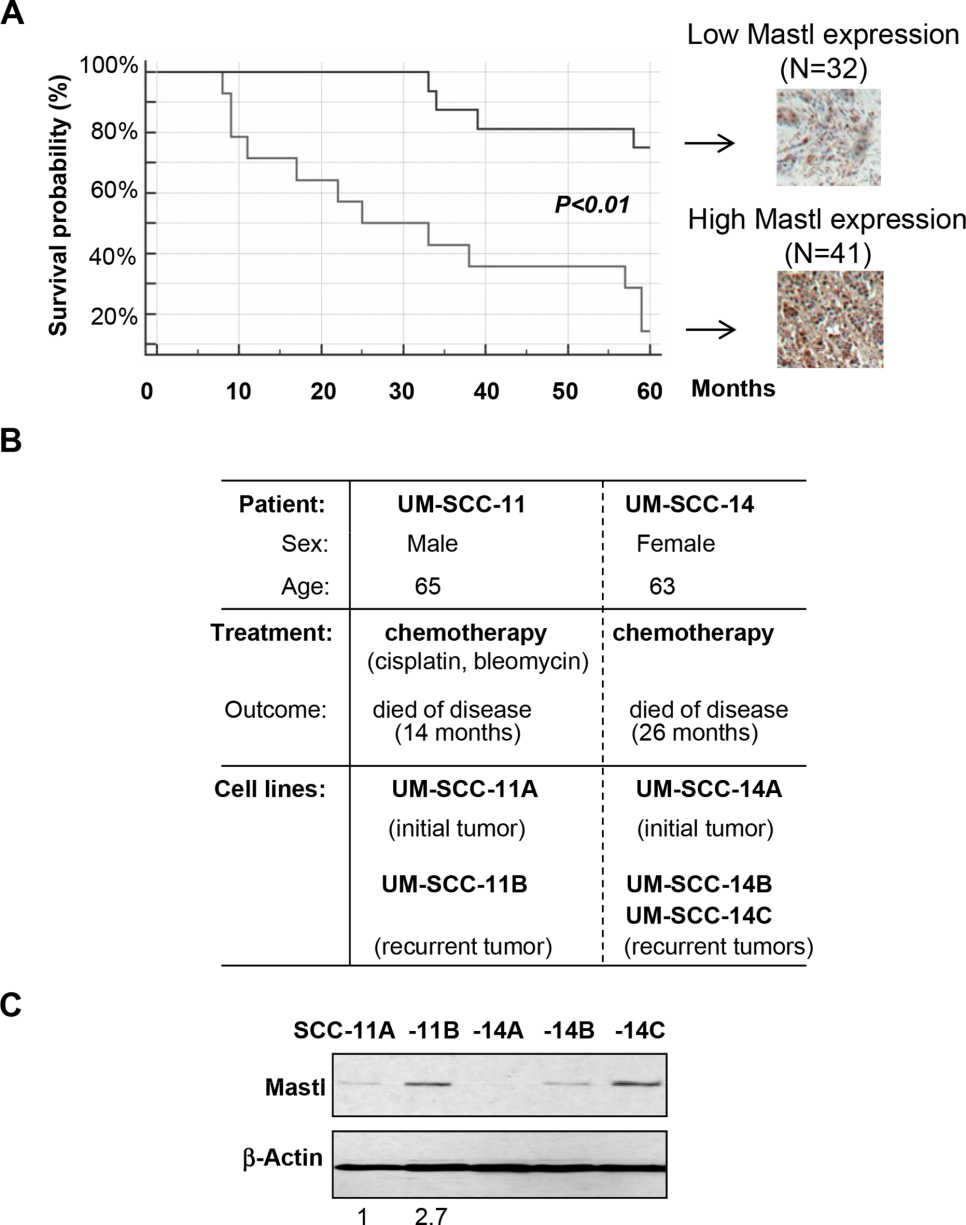
Mastl upregulation is correlated with poor patient survival and tumor recurrence **(A)** As in Fig. 1C, Mastl expression was examined in breast cancer tissues, which were then classified into two groups with either low or high Mastl expression. The survival probability is shown for patients in both groups. **(B)** The clinical information of UM-SCC-11 and UM-SCC-14 patients and cell lines is shown. **(C)** Immunoblotting of Mastl and β-Actin in UM-SCC-11 and -14 cell lines is shown. The band intensity was measured and the Mastl/β-Actin ratio shown below.

We sought to investigate whether Mastl upregulation could influence the outcome of cancer treatment. Although we currently do not have enough information on the treatment follow-up for the oral cancer cases, our analyses of breast cancer tissues showed a significant correlation between Mastl expression and poor patient survival (Fig. 2A). The study thus revealed an important role of Mastl in cancer, and suggested that Mastl expression may serve as a diagnostic and prognostic marker.

As we discovered that Mastl suppresses the DDR and promotes checkpoint recovery [19], we hypothesized that Mastl may play a role in tumor recurrence after chemotherapy. A critical finding was that Mastl upregulation accompanied tumor recurrence in both of the two recurrent head and neck squamous cell carcinoma cases examined (Fig. 2B & 2C). The UM-SCC-11A, -11B, -14A, -14B, and -14C cell lines utilized in this study were derived from tumors of two patients before and after chemotherapy using cisplatin and other agents (Fig. 2B) [33].

### Mastl upregulation promotes cell proliferation under the stress condition

**Figure 3:**
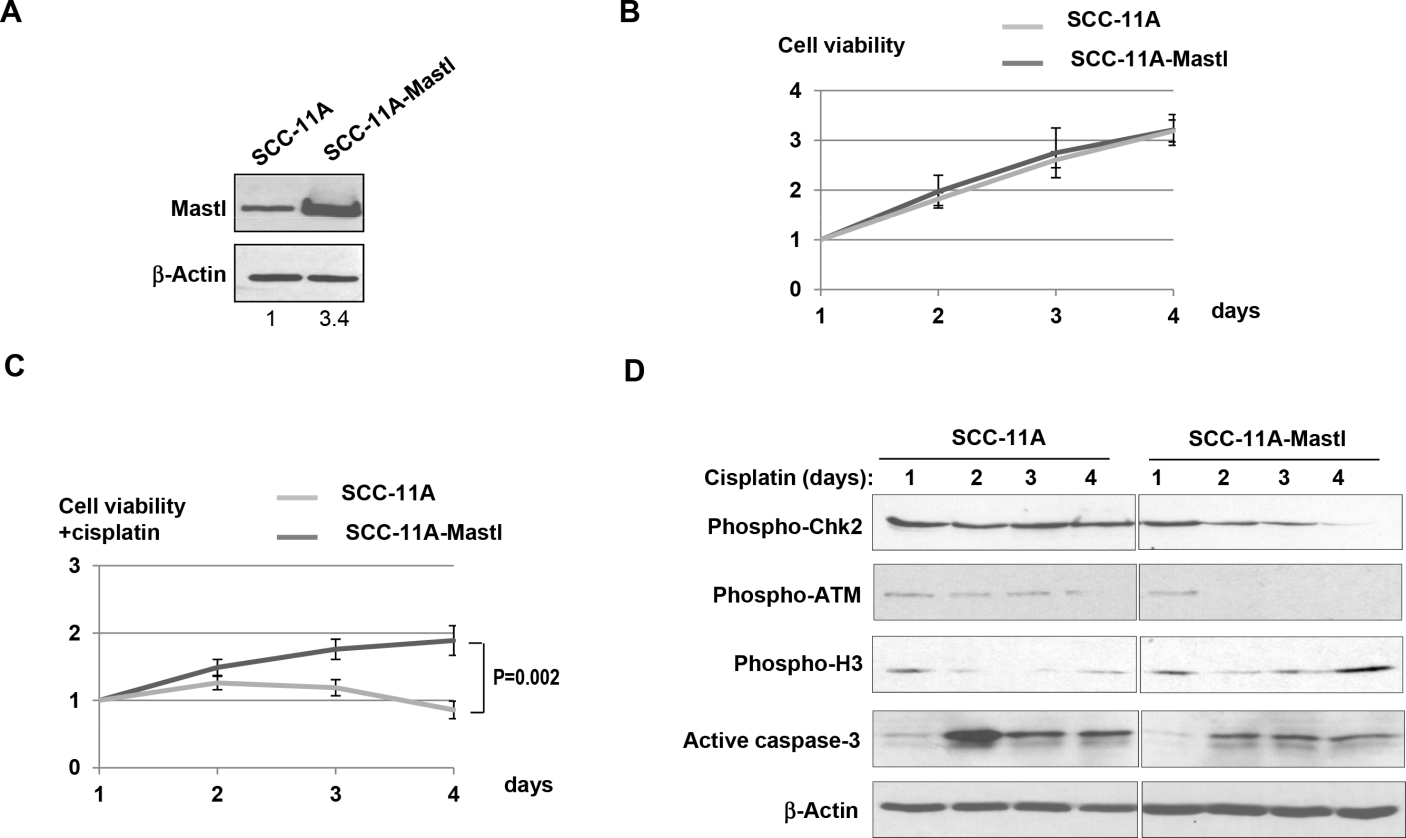
Mastl upregulation promotes cell proliferation under DNA damage stress **(A)** UM-SCC-11A and SCC-11A-Mastl cells were analyzed by immunoblotting for Mastl and β-Actin. The band intensity was measured and the Mastl/β-Actin ratio shown below. **(B)** The number of UM-SCC-11A and SCC-11A-Mastl cells was measured for 4 days. The cell number of each day was normalized to that of the first day for the relative cell viability. **(C)** The number of UM-SCC-11A and SCC-11A-Mastl cells treated with cisplatin (3.3 μM) was measured for 4 days. The cell number of each day was normalized to that of the first day for the relative cell viability. **(D)** UM-SCC-11A and SCC-11A-Mastl cells with or without treatment with cisplatin (3.3 μM) were analyzed by immunoblotting for phospho-Chk2, phospho-ATM, phospho-H3, active caspase-3, and β-Actin.

As described in Fig. 2B, UM-SCC-11A cells were derived from the original head and neck tumor. The patient was then treated with cisplatin and other therapeutic agents, but experienced tumor recurrence, and subsequently died of the disease. UM-SCC-11B cells derived from the recurrent tumor exhibited elevated expression of Mastl. To recapitulate the oncogenic upregulation of Mastl and examine its impact on cancer progression and resistance, we ectopically and stably expressed Mastl in human UM-SCC-11A cells using a retrovial vector. The resulted SCC-11A-Mastl cells expressed Mastl to approximately 3-fold over the endogenous level (Fig. 3A).

We examined the influence of Mastl upregulation on cell proliferation. To our surprise, SCC-11A-Mastl cells did not exhibit a significantly higher rate of cell proliferation compared to the control UM-SCC-11A cells (Fig. 3B). However, cell proliferation in the presence of cisplatin was significantly increased in SCC-11A-Mastl (Fig. 3C). Therefore, upregulation of Mastl led to proliferative advantage under stress conditions. Cisplatin causes DNA damage which subsequently leads to activation of both the checkpoint that halts cell cycle progression, and apoptosis that eliminates the damaged cell. We examined the phosphorylation of ATM and Chk2 as markers of the DNA damage-induced checkpoint signaling, phosphorylation of histone H3 as a marker of cell proliferation, and activation of caspase-3 as an indication of apoptosis. SCC-11A-Mastl cells treated with cisplatin exhibited reduced and less-sustained checkpoint signaling and apoptosis, compared to SCC-11A cells under the same condition (Fig. 3D). We also observed in these cells an increased level of H3 phosphorylation (Fig. 3D). Collectively, these lines of evidence suggest that Mastl influences cell proliferation under stress conditions by both suppressing cell death and promoting cell proliferation.

### Downregulation of Mastl re-sensitized the recurrent tumor cells to cisplatin

**Figure 4:**
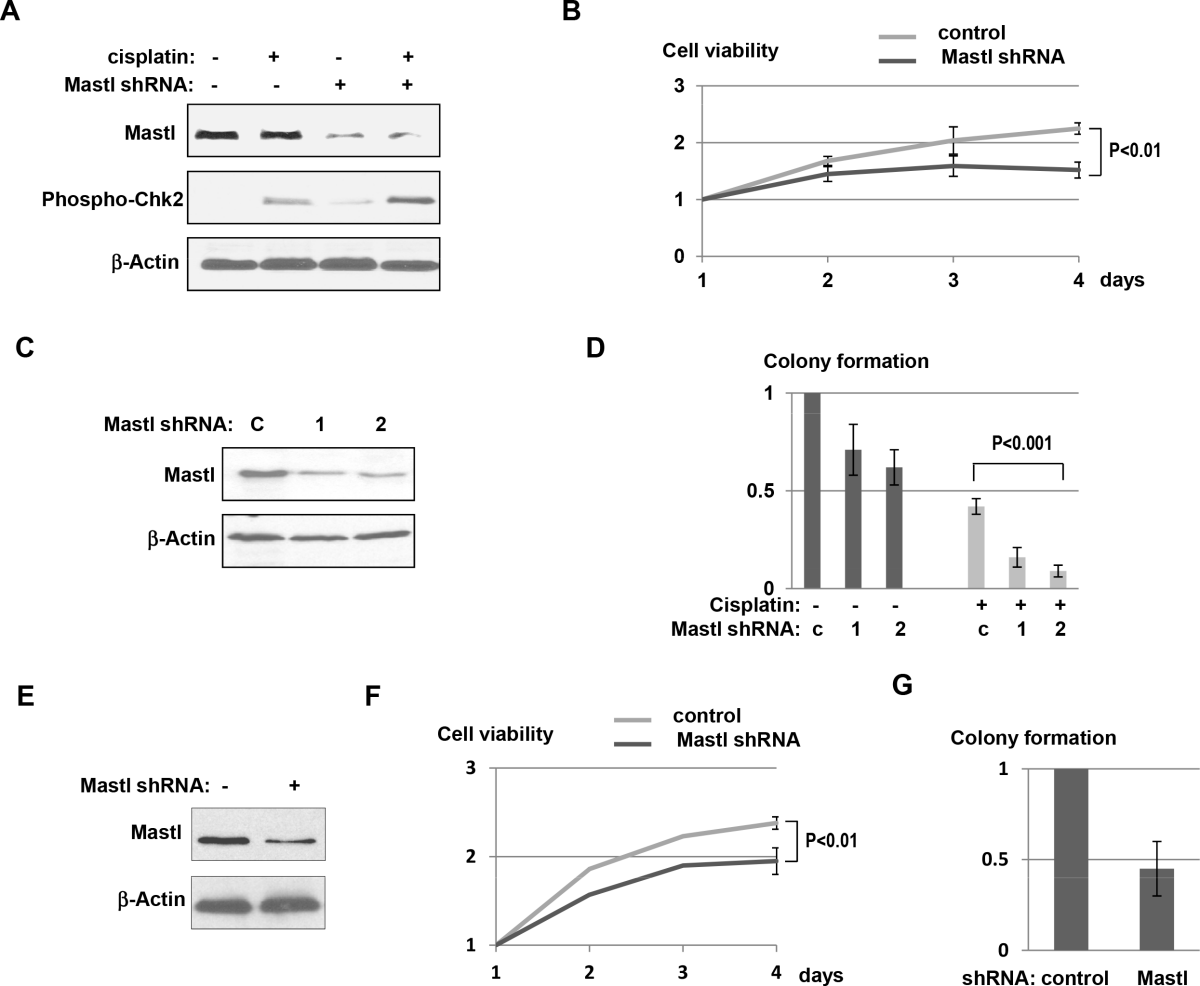
Mastl knockdown sensitized resistant tumor cells to cisplatin **(A)** UM-SCC-11B cells were treated with control, non-targeting or Mastl shRNA lentiviral particles (multiplicity of infection: 5). Two days post infection, these cells were treated with or without cisplatin (3.3 μM) for 12 hr, harvested and analyzed by immunoblotting for Mastl, phospho-Chk2, and β-Actin. **(B)** UM-SCC-11B cells with or without treatment of Mastl shRNA lentiviral particles were incubated in cisplatin (3.3 μM) for 4 days. Relative cell viability was determined as in Fig. 3B. **(C)** UM-SCC-11B cells infected with control or Mastl shRNA lentiviral particles (#1 and #2) were selected with puromycin for clones that stably expressed shRNA. Cells were then harvested and analyzed by immunoblotting for Mastl and β-Actin. **(D)** UM-SCC-11B cells with control or Mastl shRNA (as in panel C) were cultured with or without cisplatin. Clonogenic assay was performed as described in Materials and Methods. **(E)** UM-SCC-14C cells were treated with control or Mastl shRNA lentiviral particles as in panel A. Cells were analyzed by immunoblotting for Mastl and β-Actin. **(F)** UM-SCC-14C cells with or without Mastl shRNA lentiviral particles, as in panel E, were incubated in cisplatin (3.3 μM) for 4 days. The relative cell viability is shown. **(G)** The clonogenic assay was performed using UM-SCC-14C cells with or without Mastl knockdown, in the presence of cisplatin, as in panel D.

To assess the functional relevance of Mastl upregulation in UM-SCC-11B cells, we reduced its expression using a lentivirus-based shRNA vector. These cells with Mastl knockdown exhibited a detectable level of Chk2 phosphorylation even without induction of exogenous DNA damage, suggesting that a high level of Mastl expression is required to suppress activation of endogenous DNA damage signaling in UM-SCC-11B cells (Fig. 4A). Moreover, Mastl knockdown enhanced Chk2 phosphorylation in response to cisplatin (Fig. 4A), and reduced cell viability in the presence of cisplatin (Fig. 4B). These results indicated that Mastl knockdown effectively sensitized the resistant UM-SCC-11B cells to cisplatin. To further validate this conclusion, we derived from UM-SCC-11B two clones that stably expressed two different lentiviral Mastl shRNA vectors. (Fig. 4C). The long-term effect of Mastl suppression on the proliferation and resistance of UM-SCC-11B was examined using a clonogenic assay. Downregulation of Mastl led to a moderate decrease of colony formation in UM-SCC-11B cells, suggesting a partial dependence of these cells on the high-level expression of Mastl (Fig. 4D). Importantly, colony formation of UM-SCC-11B cells in the presence of cisplatin was greatly reduced by Mastl knockdown (Fig. 4D). We then further confirmed the effect of Mastl knockdown using another recurrent head and neck squamous cell carcinoma cell line, UM-SCC-14C (Fig. 4E). Mastl knockdown in UM-SCC-14C reduced both the cell viability and colony formation after cisplatin treatment (Fig. 4F & 4G). These results, collectively, demonstrated the crucial role of Mastl in the proliferation and resistance of recurrent tumor cells.

### Mastl knockdown enhances tumor response *in vivo*

**Figure 5:**
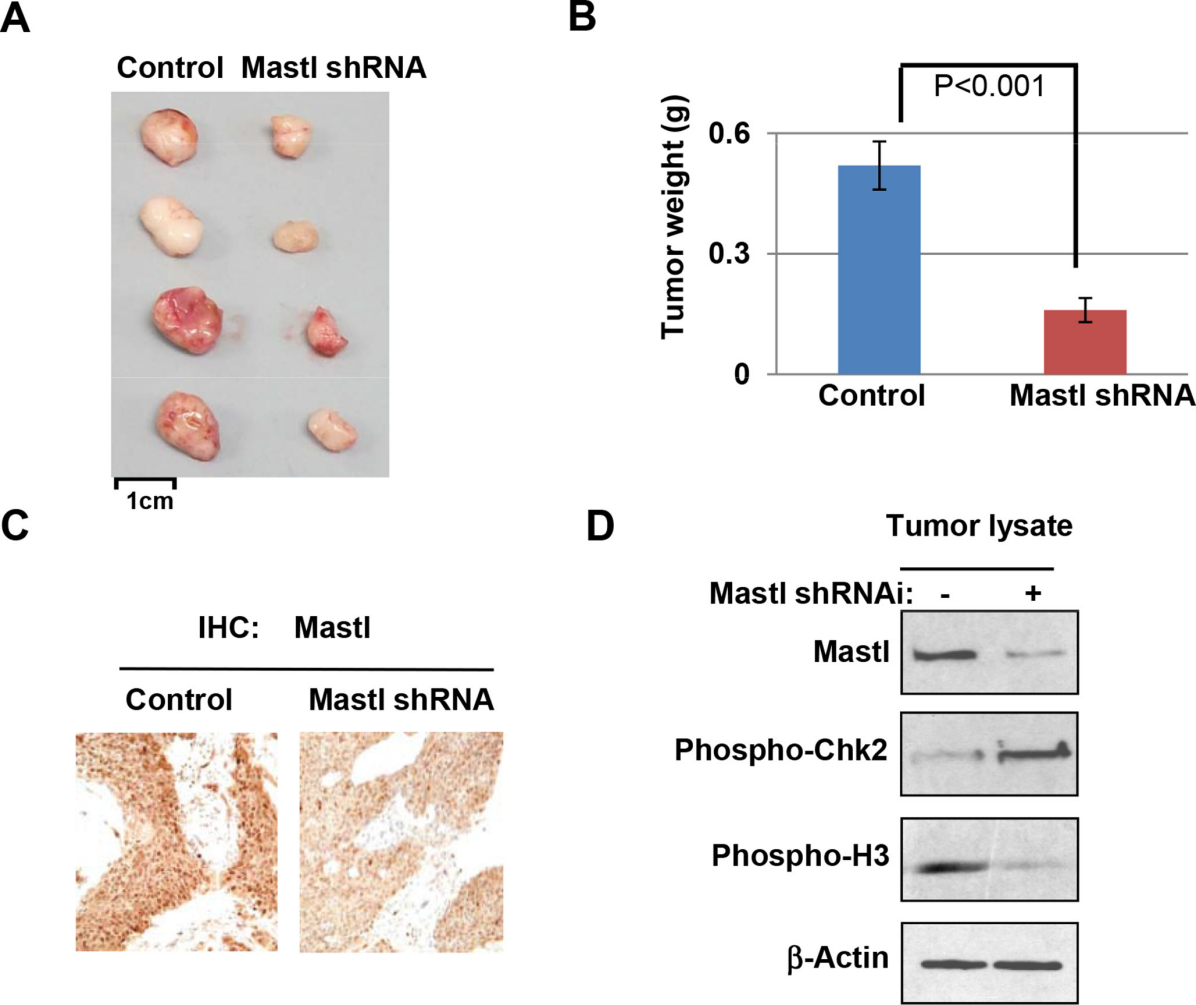
Targeting Mastl *in vivo* **(A)** UM-SCC-11B cells with control or Mastl shRNA (Fig. 4C, #2) were implanted into immunodeficient mice to form subcutaneous tumors. Once the volume of each tumor reached 50 mm^3^, the host mouse was administered with cisplatin (5 mg/kg) intraperitoneally every day for 5 days. The tumors were excised 10 days after the initial treatment. **(B)** Tumors in panel A were excised and weighted. The average tumor weight and statistical significance is shown (N = 4). **(C)** Representative tumors in panel A were analyzed by immunohistochemistry for Mastl expression. **(D)** Representative tumors in panel A were analyzed by immunoblotting for Mastl, phospho-Chk2, phospho-Histone H3, and β-Actin.

The role of Mastl overexpression in cancer was further examined using a xenograft tumor model. Implantation of UM-SCC-11B cells yielded growth of subcutaneous tumors in immunodeficient mice. Mastl-knockdown did not prevent or significantly retard the tumor growth of UM-SCC-11B cells (data not shown), suggesting that a partial reduction of Mastl alone was not sufficient to suppress the *in vivo* progression of these established cancer cells, at least in the current xenograft assay with simultaneous injection of a large number of cancer cells. We then sought to evaluate the potential of Mastl targeting in combination with cisplatin, an existing treatment option for oral cancer and several other types of cancer. We first allowed tumors to reach approximately 50 mm^3^ in volume, and then administered cisplatin (5 mg/kg) intraperitoneally every day for 5 days. The tumors were excised 10 days after treatment (Fig. 5A), and weighed (Fig. 5B). Our results showed an enhanced tumor response to cisplatin with Mastl knockdown, and thereby validated Mastl as a promising target for cancer therapy. Pathological and biochemical analyses of these tumors confirmed the knockdown of Mastl (Fig. 5C & 5D). Consistent with our studies using cultured cells (Fig. 3 & 4), tumors with Mastl knockdown exhibited increased DDR signaling, as judged by Chk2 phosphorylation, and decreased cell proliferation, as judged by mitotic phosphorylation of histone H3 (Fig. 5D).

### Mastl targeting specifically potentiates cancer cells to chemotherapy while sparing normal cells

**Figure 6:**
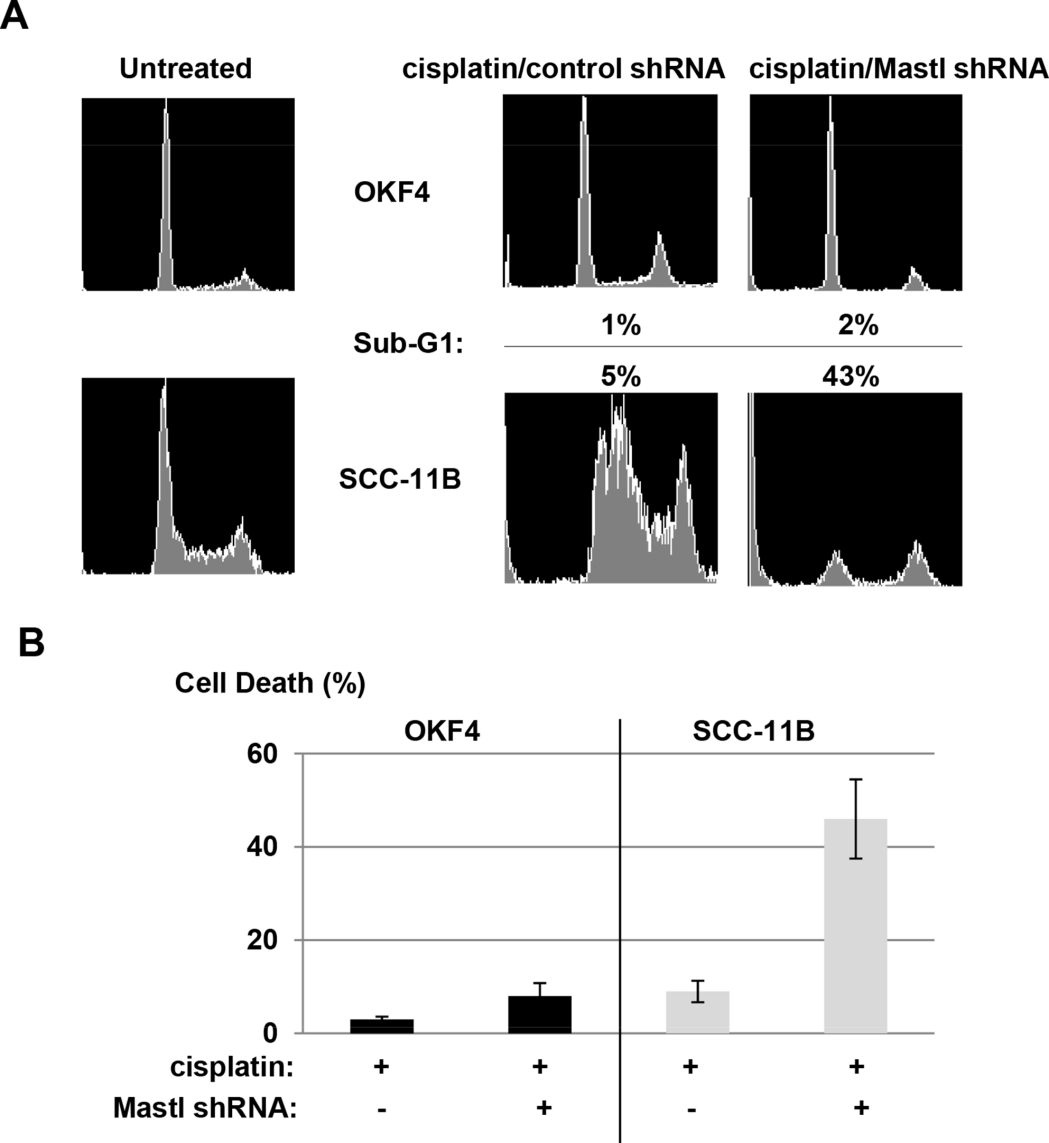
Targeting Mastl leads to specific chemosensitization in cancer cells **(A)** OKF4 or UM-SCC-11B cells were treated with cisplatin (7.8 μM) and lentiviral shRNA as indicated. 24 hours after the treatment, cells were analyzed by flow cytometry. The sub-G1 population is indicated. **(B)** OKF4 and UM-SCC-11B cells were treated as in panel A, and measured by trypan blue excursion assay for cell death.

With the validation of Mastl as an effective target to sensitize UM-SCC-11B cells to cancer therapy, it is important to investigate the effect of this treatment in normal cells. Using flow cytometry, we showed that Mastl knockdown in UM-SCC-11B cells led to greatly increased sub-G1 population after cisplatin treatment (Fig. 6A), suggesting efficient induction of apoptosis. Interestingly, the same treatment in normal oral keratinocyte OKF4 cells did not cause significant cell death (Fig. 6A). We also measured cell death using a trypan blue exclusion assay, in which Mastl knockdown sensitized cisplatin-induced death in SCC-11B but not OKF4 cells (Fig. 6B).

## DISCUSSION

The function of Mastl as an essential mitotic kinase has been extensively characterized in *Drosophila*, *Xenopus*, and mammalian cells. Our recent studies also indicated that Mastl plays a role in regulation of the DDR in *Xenopus* egg extracts [19]. In this study we showed that Mastl may act as a new oncogene in light of several lines of evidence. First, the upregulation of Mastl was noted in a substantial portion of head and neck cancer cell lines, oral squamous cell carcinoma, breast cancer, and prostate cancer tissues. Mastl upregulation correlated with aggressive clinicopathological features in the examined cancer specimens. These results suggest a role for Mastl in promoting cancer progression, and the potential utilization of Mastl expression as a diagnostic marker of cancer. Moreover, ectopic expression of Mastl significantly stimulated cell proliferation under cisplatin-induced stress conditions. The stress-resistant cell proliferation in cells with Mastl upregulation correlated with attenuated DNA damage signaling and apoptotic response. This finding revealed potential mechanistic insights into the oncogenic role of Mastl. It has been well-established that the DNA damage checkpoint functions as an anti-cancer barrier at the early stage of cancer progression [25–28]. Therefore, Mastl upregulation may represent a common mechanism for cancer cells to escape from the cellular surveillance. As cancer cells generally contain elevated levels of endogenous DNA damage, being able to support cell proliferation in the presence of DNA damage may be important in all stages of tumorigenesis.

A potential link between Mastl upregulation and cancer therapy was suggested as Mastl upregulation in breast cancer patients correlated strongly with poor patient survival. Because cancer treatment relies heavily on DNA damaging agents, including radiation and chemotherapeutic drugs, we hypothesized that Mastl upregulation renders cancer cells resistant to treatment, and thereby, increases the risk of tumor recurrence and patient mortality. Indeed, tumor resistance and recurrence stands as a major challenge to cancer therapy. Despite the initial success of cancer treatment in many patents, their tumors may recur and further progress, eventually leading to treatment failure and patient mortality. Thus, better understanding of the underlying molecular mechanism of tumor recurrence is imperative in predicting treatment outcome and developing new therapeutics. A critical finding that implicated Mastl upregulation to tumor recurrence was obtained from two clinical head and neck cancer cases. In both cases, tumors recurred after cisplatin and other treatments. Interestingly, comparative analysis using cell lines derived from the original and recurrent tumors revealed strong upregulation of Mastl in the recurrent tumor cells. The notion that upregulation of Mastl contributed to the recurrence of the head and neck tumors was extensively analyzed in this study. Knockdown of Mastl in the recurrent tumor cells led to re-sensitization of these cells to cisplatin, as judged by decreased cell proliferation and increased DNA damage signaling. The role of Mastl was further confirmed using an *in vivo* tumor model that illustrated an enhanced tumor response to cisplatin with Mastl knockdown.

Our results demonstrated the involvement of Mastl upregulation in cancer progression and its potential value in the prediction of treatment response and patient survival. Furthermore, this study validated Mastl as a promising target for cancer therapy. This information may be of direct clinical value as protein kinases are known to be highly susceptible for pharmacological targeting. Our results in cell lines and xenograft tumor models revealed that targeting Mastl re-sensitized the resistant head and neck cancer cells to cisplatin. The finding is interesting because these cancer cells were derived from recurrent tumors which survived the initial treatment with cisplatin and other chemotherapeutics. We speculated that in tumor cells harboring Mastl overexpression, it is beneficial, and perhaps necessary to disrupt Mastl function in order to achieve an ideal therapeutic outcome. The study thus calls for immediate research efforts in the characterization of kinase inhibitors that specifically target Mastl, and validation of these inhibitors for cancer therapy. Considering that Mastl upregulation in a substantial portion of cancer cells and tissues, the therapeutic strategy of targeting Mastl may yield broad potential in the treatment of various types of cancer.

An ideal strategy for cancer therapy is to exploit the difference between cancer and normal cells, and develop treatments that confer specific toxicity to cancer cells while sparing normal cells. If tumors rely on Mastl upregulation to progress and escape cancer therapy, then targeting Mastl function can be a specific way to improve cancer therapy and prevent disease relapse. Importantly, Mastl knockdown in combination with cisplatin treatment induced substantial cell death in resistant tumor cells, but not in non-tumorigenic oral keratinocyte cells. While the cancer-specific sensitization of chemotherapy by Mastl needs to be further investigated, a few possibilities ought to be considered. First, even though Mastl plays important roles in the basic cell cycle machinery, non-tumorigenic cells, such as OKF4 and HaCaT cells, can proliferate with relatively low levels of Mastl, suggesting an optimal therapeutic window. A recent study showed that cells with complete knockout of Mastl still entered mitosis [18]. Second, normal cells may be protected from cell death by the p53-dependent checkpoint. For example, the immortalized oral keratinocyte cells used in this study are known to possess intact G1 checkpoint arrest via p53 activation [34], whereas the mechanism is often crippled in cancer cells, such as UM-SCC-11B [32]. Third, cancer cells often contain higher levels of endogenous DNA damage, due to DNA repair deficiencies, oncogene-induced DNA replication, and other features associated with cancer progression. Therefore, cancer cells can be particularly addictive to upregulation of Mastl to survive and proliferate. Future studies are necessary to better characterize the role of Mastl in cancer progression and recurrence, as well as to investigate the clinical potential of Mastl inhibition for cancer therapy.

## MATERIALS AND METHODS

### Cell culture and analysis

Human oral/laryngeal squamous-cell carcinoma cell lines were obtained from Dr. Thomas Carey (University of Michigan) in 2010 and 2011. These cell lines were previously characterized genetically and morphologically [33, 35]. Cells were maintained in Dulbecco's modified Eagle medium (DMEM, Sigma, St Louis, MO) supplemented with 10% fetal bovine serum (FBS, Sigma). To measure cell sensitivity to cisplatin, cells were treated with cisplatin at indicated concentrations, and incubated for 1–4 days. The numbers of viable cells were counted using a hemocytometer. Lentiviral vectors expressing control non-targeting or Mastl shRNAs were purchased from Sigma and used to infect cells following the protocol recommended by the manufacturer. The effect of cisplatin and Mastl knockdown on the survival and proliferation of UM-SCC-11B cells was determined by clonogenic assay, as described in a previous study [35]. Briefly, cells were seeded into 6-well plates at a density of 1,000 cells per well. After 24 hours, cells were treated with or without cisplatin. After incubation for 2 weeks, cells were then fixed in 1% glutaraldehyde for 30 minutes, stained with 5% crystal violet, and counted for colony numbers. Cell cycle progression of UM-SCC-11B and OKF4 cells was examined by fluorescent-activated cell sorting flow cytometer (FACS), as described in a previous study [36]. Briefly, cells were fixed in phosphate buffered saline (PBS) buffer with 4% formaldehyde, washed, and incubated in 50 μg/ml propidium iodide and 100 μg/ml RNase A for 30 min, and 10,000 cells per sample were analyzed on a BD FACSarray (BD Biosciences). Trypan blue staining was performed by mixing 0.4% trypan blue in PBS with cell suspension at a 1:10 ratio.

### Immunoblotting

Immunoblotting was performed as described previously [31]. Anti-phospho-Chk2 Thr-68, and phospho-ATM Ser-1981 antibodies were purchased from Cell Signaling (Danvers, MA); anti-phospho-H3 Ser-10, active caspase-3, β-Actin antibodies were obtained from Abcam (Cambridge, MA). Monoclonal antibody to Mastl (clone 4F9, Millipore) was generated against the C-terminus of Mastl. The intensity of band signals was measured using NIH Image-J software.

### Immunohistochemistry

The slides were deparaffinized with immersion in xylene and rehydrated in alcohol of sequentially decreasing concentrations, and then autoclaved for 7 minutes in Tris-EDTA buffer pH 9.0 (10 mM Tris Base, 1 mM EDTA, 0.5 mL Tween 20, pH 9.0) for antigen retrieval. After treatment with 3% hydrogen peroxide solution to block endogenous peroxidase activity for 10 minutes, slides were blocked with 10% normal goat serum in PBS for 1 hour at room temperature. Slides were subsequently incubated with primary antibody at 4°C overnight. Biotinylated secondary antibody (BD Pharmingen) was applied to slides at 1:100 dilution for 1 hour. Bound antibody was detected with a streptavidin-biotin system. For color development, slides were incubated with DAB substrate solution for 45 seconds and rinsed in water three times for 4 minutes. Following hematoxylin counterstain for 5 minutes, slides were dehydrated in alcohol and xylene and a coverslip was mounted over each slide. Negative control slides were processed without primary antibody. Paraffin-embedded oral tissue samples were obtained from the Oral Pathology biopsy service at the University of Nebraska Medical Center (UNMC) College of Dentistry and from the Department of Pathology and Microbiology at the UNMC College of Medicine. Institutional review board (IRB, #625–11) approval was obtained for the use of human tissues for this study. The Imgenex Histo-Array and US Biomax tissue array slides consisted of breast, prostate cancer tissues and control tissues were similarly examined by immunohistochemistry for Mastl expression. Staining of all slides was evaluated independently by two observers. Based on the intensity of Mastl staining, the tissue slides were designated as either weak = low expression (generally at a comparable level as in the control, normal tissues), or strong = high expression. Evaluation of the samples was performed under the supervision of a board-certified oral and maxillofacial pathologist (P.G.). Statistical significance was analyzed using an unpaired 2-tailed Student's t-test.

### Xenograft tumor model

Athymic nude mice were purchased from NIH and housed at the animal facility at the UNMC College of Dentistry. SCC cells were implanted into 6–week old female mice by a single subcutaneous injection of tumor cells (2 - 6 × 10^5^ cells in 100 microliters of sterile PBS). To test how tumors respond to chemotherapy, once the tumor size reached 50 mm^3^, cisplatin (5 mg/kg mouse) was administered intraperitoneally every day for 5 days. Ten days after the initial treatment, the mice were euthanized, and tumors were removed and weighed. The volume of the tumor was compared among all experimental groups. Data were analyzed using an unpaired 2-tailed Student's t-test to determine the statistical significance.
